# A new tool to quantify biodiversity change under landscape transformation

**DOI:** 10.1002/eap.3071

**Published:** 2024-12-05

**Authors:** Renato Richard Hilário, William Douglas Carvalho, Bruna Da Silva Xavier, Jorge M. Palmeirim, Marcus Vinícius Vieira, Karen Mustin, Pedro Cardoso

**Affiliations:** ^1^ Department of Environment and Development Federal University of Amapá Macapá Brazil; ^2^ Post‐Graduate Program in Tropical Biodiversity Federal University of Amapá Macapá Brazil; ^3^ Terrestrial Ecology Group (TEG‐UAM), Department of Ecology, Faculty of Sciences Autonomous University of Madrid Madrid Spain; ^4^ Centro de Investigación en Biodiversidad y Cambio Global (CIBC‐UAM) Universidad Autónoma de Madrid Madrid Spain; ^5^ Post‐Graduate Program in Ecology Federal University of Rio de Janeiro Rio de Janeiro Brazil; ^6^ Center for Ecology, Evolution and Environmental Change CE3C and CHANGE – Global Change and Sustainability Institute, Faculty of Sciences University of Lisbon Lisbon Portugal; ^7^ Department of Biodiversity, Ecology and Evolution Complutense University of Madrid, Ciudad Universitaria Madrid Spain; ^8^ Laboratory for Integrative Biodiversity Research (LIBRe), Finnish Museum of Natural History (LUOMUS) University of Helsinki Helsinki Finland

**Keywords:** functional diversity, Hill numbers, land use changes, phylogenetic diversity, species diversity, taxonomic diversity

## Abstract

Identifying how species richness or diversity changes with different proportions of natural and anthropized environments in the landscape is important for landscape management for conservation. Here, we propose a new method to assess biodiversity changes in landscapes with varying proportions of habitat types. The algorithm is based on the resampling of individuals recorded in different habitats considering both the proportion occupied by each habitat in the landscape and the number of individuals recorded in each habitat. The diversity is assessed based on the sampled individuals. If a functional/phylogenetic tree or distance matrix is provided, the function returns the functional or phylogenetic richness values. This procedure is replicated a number of times with different proportions of each of the habitats in the landscape. Our method copes with two or more habitat types in the landscape and works with taxonomic, functional, and phylogenetic diversities. We tested our method using 10 different simulated scenarios and one empirical dataset with bats (Chiroptera) to assess whether they behaved as expected. Our method performed as expected in all scenarios and in the empirical dataset (considering also the functional and phylogenetic diversities in this latter case). The possibility of working with more than two habitat types and with different dimensions of diversity (i.e., functional and phylogenetic diversity) is a major advantage of the new method. We show that this is a valuable tool to assess biodiversity changes in the context of landscape planning, helping to promote more sustainable landscapes often composed of multiple habitat types with mixed biodiversity composition.

## INTRODUCTION

Land use change, which includes the substitution of native habitats with anthropogenic environments, is among the primary factors that threaten global biodiversity (Thomas, 2020). Different species respond differently to environmental changes, with some species disappearing or reducing their abundance in anthropogenic environments, while others thrive in such environments (Joyce et al., 2018; Stork et al., 2009). The fact that some species are limited to natural habitats while others are mostly observed in anthropogenic environments can often lead to increased species richness (or diversity) in landscapes with intermediate amounts of each type of environment (Thomas, 2020). Therefore, identifying the proportion of each environment that maximizes species richness (or diversity) in the landscape is important to project and manage landscapes that conserve biodiversity and ecosystem services in particular, which are essential for human well‐being and economic prosperity. Although maximizing species diversity in the landscape may be a common conservation goal, it is important to highlight that conservationists should exercise due caution whenever higher biodiversity in the landscape is a result of the inclusion of alien species in a landscape.

There have been substantial recent advances in the development of richness and diversity estimators. Jost (2006) reintroduced the use of Hill numbers to estimate richness and diversity in an integrated framework. In this framework, diversity is assessed by 
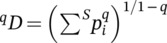
, where $p_{i}$ is the relative abundance of the *i*th species in the assemblage and *S* is the number of the species in the assemblage. The parameter *q* represents the sensitivity of the estimation to relative species frequencies (Chao, Gotelli, et al., 2014). When *q* = 0 (i.e., abundances are not considered), the index matches species richness; when *q* = 1, it corresponds to the exponent of the Shannon's diversity index; and when *q* = 2, it relates to the Simpson's diversity index (Chao, Gotelli, et al., 2014; Jost, 2006). Therefore, the higher the value of *q*, the higher the weight given to abundant species, which are often the main ecosystem components (Chao, Gotelli, et al., 2014). There have also been advances in the integration of functional and phylogenetic diversity under multiple unified frameworks, each one with particular strengths and weaknesses (Chao, Chiu, & Jost, 2014; Mammola et al., 2021). Functional diversity is based on the diversity of traits that impact fitness indirectly via its effects on growth, reproduction, and survival (Violle et al., 2007). Likewise, phylogenetic diversity reflects the evolutionary history and relationships between species composing a community or assemblage. Both functional and phylogenetic structures can be incorporated in the calculation of diversity through trees that represent the functional or phylogenetic distances between species (Cardoso, Guillerme, et al., 2024). In this framework, taxonomic diversity can be implemented similarly with a tree in which all species are linked directly to a central node through a unit length branch, ensuring comparability between these different diversity types (Cardoso, Guillerme, et al., 2024).

Recently, Chao et al. (2019) proposed a way to combine richness/diversity curves from two different habitat types, which allows the estimation of richness/diversity in landscapes with different proportions of each habitat type. This approach is based on samples of the species assemblages taken from two different habitats. It assesses the frequency of each species in a mixed sample of the two habitats, considering the proportion of these habitats in a landscape. To obtain the mixed sample, a proportion of the individuals in the original habitat is replaced by the same number of individuals of the transformed habitat. Then, the species richness/diversity of the combined sample is calculated based on the joint abundance frequency count of all species in the samples. This procedure is carried out with different proportions of the two habitats in the landscape, ranging from 100% of the original habitat to 100% of the transformed habitat. Note that we use the terms original and transformed habitat following Chao et al. (2019), although this approach can be applied to any pair of habitats, regardless of whether they are original or not.

The method proposed by Chao et al. (2019) represents a major conceptual advance in the way we measure biodiversity change when the landscape is transformed and is intended to estimate the resulting diversity of the landscape when a proportion of a habitat is converted into another habitat with a standardized sample size. Therefore, this method replaces a number of individuals in one habitat by the same number of individuals in the other habitat. Considering that habitat conversion may affect the number of individuals found in the landscape (i.e., carrying capacity), a method intended in identifying the proportion of each habitat in the landscape that maximizes the gamma diversity needs to consider the variation in the carrying capacity. In addition, landscapes are frequently composed of more than two habitat types. Also, ecologists may be interested in examining how landscape changes may affect the diversity of life forms and interactions (functional diversity) or the number of lineages (phylogenetic diversity) in the landscape.

Here, we propose a new method to quantify several facets of biodiversity with different proportions of multiple habitat types and this way identify the proportions that maximize biodiversity. Our method can deal with two or more types of habitats in the landscape, and in addition to taxonomic diversity, it allows the estimation of functional and phylogenetic diversities, which are important to infer about the loss of unique life forms or lineages in the landscape, respectively. Furthermore, our method considers the change in the number of individuals in different habitat types, which may lead to more accurate estimates for most scenarios where carrying capacity is affected by habitat transformation. To test our proposed method, we use data from both simulated communities for which we change specific characteristics, and an empirical example using tropical bats, and evaluate whether the method performs according to our expectations.

## METHODS

### The method (mixture)

The algorithm proposed here, named “mixture,” is based on the resampling of individuals recorded in different habitats, conditioned on the observed relative abundance of individuals in each habitat. The total number of individuals in the landscape may change, among other factors, as a consequence of habitat conversion. The algorithm “mixture” works as follows. Suppose that we have sampled a species assemblage ($a_{h}$) associated with a habitat (h) and recorded $n_{h}$ individuals belonging to $s_{h}$ species. The observed frequencies of each species in this sample are given by the vector $X_{h}=\left(X_{1h},X_{2h},X_{3h},\ldots X_{\mathrm{sh}}\right)$. In a landscape with i habitat types and i correspondent species assemblages, we expect differences in the number of individuals sampled in each of these assemblages. Therefore, to estimate the pooled species richness/diversity (^*q*^ *D*) in a landscape with different proportions of these habitats, we must combine these habitats' assemblages considering both the proportion occupied by each habitat in the landscape and the number of individuals recorded in each habitat. The proportion of habitat h in the landscape is $p_{h}$, and $\sum_{h=1}^{i}p_{h}=1$ with i being the number of habitat types. Thus, by resampling $m_{h}=p_{h}\times n_{h}$ individuals from each habitat, we can derive a pooled assemblage ($\sum_{h=1}^{n}m_{h}$) for the whole landscape, with the probability of selecting each individual from a given habitat corresponding to their species abundances, that is, vector $X_{h}$. From this pooled assemblage, we can estimate the species diversity (^*q*^ *D*) of the whole landscape based on taxonomic (Hill numbers), phylogenetic, or functional diversity.

This procedure should be carried out with different proportions of each of the habitats ($p_{h}$) in the landscape, varying from the complete dominance of a given habitat ($p_{h}=1$) to its complete absence ($p_{h}=0$) in prespecified intervals (e.g., 0.1 intervals). Thus, we can obtain diversity estimates for all possible combinations of habitat proportions within a landscape. We can estimate SEs and CIs of ^*q*^ *D* by bootstrapping this procedure a number of times.

We developed the R function “mixture” using this procedure to estimate (^*q*^ *D*) allowing different *q* values or representations of functional and phylogenetic diversity. For taxonomic diversity, the function allows any value of *q*, although commonly used values are *q* = 0 (i.e., species richness), *q* = 1 (i.e., the exponential Shannon's diversity index), and *q* = 2 (i.e., the Simpson's diversity index). The phylogenetic and functional diversities are calculated only for *q* = 0. The function allows changing CI levels and the number of bootstrap runs, and choosing the increment of the sequence of $p_{h}$ (i.e., the intervals in which the proportion of each habitat changes). The function also allows resampling the assemblages with or without replacement, although we only recommend its use with replacement (see below). If a functional/phylogenetic tree or distance matrix is provided, the function returns the functional or phylogenetic richness values. Taxonomic diversity is assessed using the function “hill” of the package BAT (Cardoso et al., 2015) on the resampled pooled assemblages with the specified q value. The function “mixture” uses the function “alpha” of the package “BAT” to return the functional and the phylogenetic diversities considering the provided phylogenetic/functional tree or distance matrix. The function “mixture” was included in an update of the package BAT of the software R (R Core Team, 2022).

### Simulations

To assess the performance of the proposed method in different situations, we simulated 10 different scenarios. In each scenario, we created two assemblages (which are associated with two different habitats) according to some prespecified attributes and predicted how the pooled richness should vary with different proportions of these assemblages. The proportions varied from 0 to 1 in intervals of 0.1. Table 1 describes the different scenarios and their respective expectations and the created assemblages are available in Hilário (2023c). Although the function “mixture” can be used with any number of assemblages, here we used simulations with two assemblages only in order to simplify the comparison of prediction with outcome. Only Scenario 1, which has the simplest prediction, was tested with three assemblages.

**TABLE 1:** Tested scenarios and expectations of species richness and diversity with changes in the proportion of the area occupied by habitats in the landscape.

Scenario	Description	Details of how we generated the assemblages	Prediction
1	Two or three exactly equal assemblages.	The assemblages were created with 30 species and the following proportions of individuals: $X_{1},\frac{X_{1}\;}{2},\frac{X_{1}\;}{3},\frac{X_{1}\;}{4},\ldots \frac{X_{1}\;}{30}$, which is a relationship that well describes biological communities, according to Fisher et al. (1943).	The substitution of one habitat by the other should not change the species composition of the landscape. Therefore, we expect no relation between diversity and different proportions of the habitats.
2	Both assemblages have equal species richness and composition, but the species that are more or less abundant differ between assemblages.	The first assemblage is as in Scenario 1. The second assemblage is a permutation without repetition of the first assemblage.	Species richness should be greatest with intermediate amounts of each habitat, given that in this situation we should observe the dominant and rare species of both habitats. As we approach either extreme (predominance of one of the habitats), few rare species are lost, reducing the pooled species richness. This effect should increase with the value of *q*, considering that we may find a higher pooled evenness with intermediate amounts of each habitat.
3	Both assemblages have the same species richness and composition, but different evenness.	The first assemblage is as in Scenario 1. The second assemblage was created similarly, but we multiplied the species abundances (from the most dominant to the rarest species) by the following values: 3.0, 2.9, 2.8 … 0.1 and then randomized the species order.	As in the previous scenario, species richness should be greater with intermediate amounts of each habitat. However, we expect that increasing the value of *q* should lead to higher diversity in landscapes with greater amounts of the habitat with greater evenness.
4	Both assemblages have the same species richness but composition and evenness differ.	Both assemblages are as in Scenario 3. However, to each assemblage, we added five species that were absent in the other assemblage. The species order of the second assemblage was randomized.	Higher species richness and diversity should occur in landscapes with greater amounts (but not with 100%) of the habitat with greater evenness, given that this habitat should add species to the pooled assemblage faster, whereas the other habitat should add mostly individuals from a few dominant species (rare species should be observed only when this habitat reaches higher proportions in the landscape).
5	Both assemblages have the same species richness and evenness, but different species composition.	Both assemblages are as in Scenario 1. However, to each assemblage, we added five species that were absent in the other assemblage. The species order of the second assemblage was randomized.	Higher species richness and diversity should occur in landscapes with intermediate proportions of each habitat, given that differences in species composition must counterbalance each other.
6	The assemblages have different species richness.	The first assemblage is as in Scenario 1, with 30 species. The second assemblage was created with 45 species following the proportions proposed by Fisher et al. (1943) and including the 30 species of the first assemblage. The species order of the second assemblage was randomized.	We expect higher species richness and diversity in landscapes with greater amounts of the richer habitat, given that this habitat has greater richness and diversity.
7	The assemblages have different species richness, with greater abundance of individuals in the richer habitat.	Both assemblages are as in Scenario 6, but the abundance of individuals of each of the species in the second assemblage was doubled.	Higher species richness and diversity should occur in landscapes with greater amounts of the richer habitat, as the poorer habitat being fully nested within the richer. This effect should be enhanced by increasing the value of *q*, given that the poorer habitat adds mostly species with low abundance, which have lower weights when the value of *q* is higher.
8	The assemblages have different species richness, with a greater abundance of individuals in the poorer habitat.	Both assemblages are as in Scenario 6, but the abundance of individuals of each of the species in the first assemblage was tripled.	Higher species richness should occur in landscapes with greater proportions of the richer habitat, but increasing the value of *q* should lead to higher diversity in landscapes with greater amounts of the poorer habitat, given that the species contributed by the richer habitat will have a lower abundance and consequently lower weight when the value of *q* is increased.
9	The assemblages have different species richness, with the richer assemblage having an extremely dominant species.	Both assemblages are as in Scenario 6, but the most abundant species of the second assemblage (richer) had its abundance multiplied by 5.	We expect higher species richness in landscapes with greater amounts of the richer habitat, but diversity should increase with greater proportions of the poorest habitat, given that it has higher evenness.
10	The assemblages have different species richness, with the poorer assemblage having an extremely dominant species.	Both assemblages are as in Scenario 6, but the most abundant species of the first assemblage (poorer) had its abundance multiplied by 5.	Higher species richness and diversity should occur in landscapes with greater amounts of the richer habitat since it is both richer and has higher evenness.

*Note*: In the scenarios in which assemblages are different, the first assemblage corresponds to the original habitat, while the second assemblage represents the converted habitat.

### Case study

We surveyed phyllostomid bats in a landscape with a savanna matrix and patches of forest and soybean plantations in the region of the Savannas of Amapá (0°14′16.44″ N; 51°03′30.40″ W), an area of Amazonian Savanna in the northeastern extreme of the Brazilian Amazon (Mustin et al., 2017). We established seven sampling transects in forest patches and seven transects in soybean plantations (savanna areas were not surveyed). We surveyed the bats with nine mist‐nets (12 × 3 m; 14 mm mesh size) for four nights in each sampling transect, totaling 56 nights (28 nights in each habitat). On each sampling night, the bat survey lasted for 6 h starting just before sunset, with a sampling effort of 54,432 m^2^ × h for each habitat.

We captured 588 bats belonging to 33 species in forest patches and 16 bats belonging to nine species in soybean plantations. Both abundance and richness of bat species are drastically reduced in the soybean plantations as compared to the forest patches. The bat assemblages used in this study are available in Hilário (2023a).

To allow the calculation of bat functional diversity, we obtained the following species traits from different sources: log body mass (average body mass of the captured individuals, excluding pregnant females), relative wing load and wing aspect ratio (Marinello & Bernard, 2014; Tavares, 2013), trophic level (phytophagous or animalivorous; Giannini & Kalko, 2004), dietary specialization (frugivore, insectivore, omnivore, nectarivore, or sanguinivore—Ecological Register database, ecoregister.org, accessed on 15 January 2019), and vertical stratification (canopy or understory; Bernard, 2001; Kalko & Handley, 2001; Ramos Pereira et al., 2009). For more details about the species traits, see Carvalho et al. (2021). The functional distance matrix between the surveyed species was built with the function “gawdis,” which uses the Gower distance but assigns different weights to the functional traits so each trait has an equitable influence on multi‐trait dissimilarity (de Bello et al., 2021). To allow the estimation of bat phylogenetic diversity, we used a phylogenetic tree created with the function “hclust” and obtained from Upham et al. (2019). Species traits and the phylogenetic tree used in this study are available in Hilário (2023a). The proportion of forest and soybean plantations in the analysis varied from 0 to 1 in intervals of 0.1.

## RESULTS

### Simulations

**FIGURE 1:**
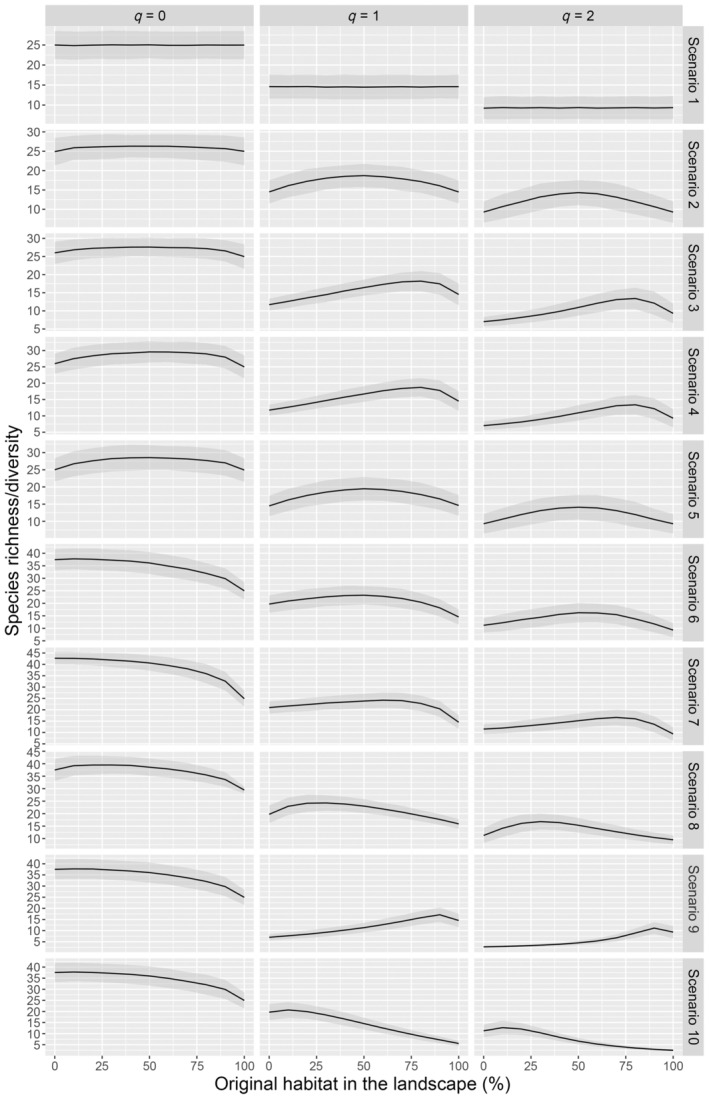
Variation of species richness/diversity according to different proportions of original habitat (which hosts the first assemblage as described in Table 1) in the landscape in the 10 tested scenarios (rows). The columns represent the results of the function “mixture” (described in this study) with replacement for *q* = 0 (species richness), *q* = 1 (exponential Shannon's diversity index), and *q* = 2 (Simpson's diversity index). The shaded areas represent the 95% CIs.

The results provided by the “mixture” function using replacement of individuals agreed with the predictions for all the scenarios when *q* = 0 (Figure 1). For *q* = 1 and *q* = 2, the results agreed with the predictions for Scenarios 1–5 and 9–10. In Scenarios 6 and 7, we predicted that higher diversity should occur in landscapes with greater amounts of the richer habitat, but for *q* = 1 and *q* = 2, the peak occurred in the middle of the interval. In Scenario 8, we predicted that increasing the value of *q* would shift the peak toward a greater proportion of the poorer habitat, but the peaks were observed in landscapes with a greater proportion of the richer habitat.

When we tested Scenario 1 with three assemblages, the results also complied with the predictions, with only slight variation in the richness/diversity for any proportion of the habitats in the landscape. For *q* = 0, the different possible combinations of landscape composition showed an average estimated richness of 25.0 ± 0.1 (mean ± SD), with a minimum of 24.8 and a maximum of 25.2. For *q* = 1, the average diversity was 14.7 ± 0.0, ranging from 14.5 to 14.7. For *q* = 2, the average diversity was 9.3 ± 0.0, ranging from 9.2 to 9.4.

### Case study

**FIGURE 2:**
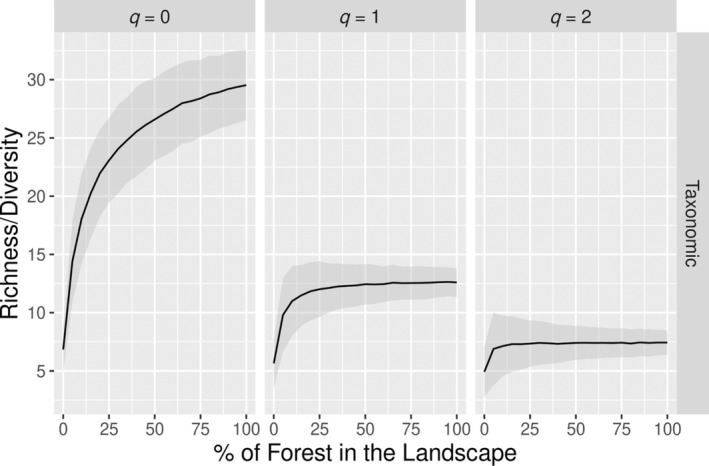
Variation of taxonomic richness/diversity according to different proportions of forest in the landscape, based on empirical data on Amazonian bat assemblages. The other habitat in the landscape is soybean plantations. Estimates were produced using the proposed “mixture” function. Columns represent the results for *q* = 0 (species richness), *q* = 1 (exponential Shannon's diversity index), and *q* = 2 (Simpson's diversity index), respectively. The shaded areas represent the 95% CIs.

**FIGURE 3:**
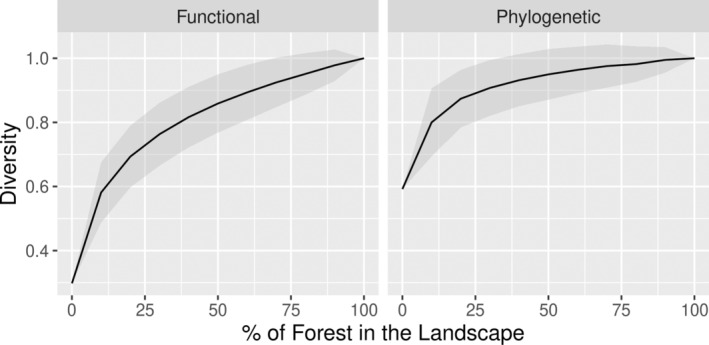
Variation of the functional and phylogenetic diversities according to different proportions of forest in the landscape, based on empirical data on Amazonian bat assemblages. Given that functional and phylogenetic diversities are unitless, we scaled the diversity values for a maximum of 1 and minimum of 0. The other habitat in the landscape is soybean plantations. The shaded areas represent the 95% CIs.

The function “mixture” indicates a maximum richness of bats in a landscape with 100% of forest for all *q* values (Figure 2), with a predictable and expected behavior across the different habitat proportions. With *q* = 1 and *q* = 2, losing some proportion of forest in the landscape does not result in significant loss of diversity, given that higher *q* values give more weight to the more common species, which tend to persist in the landscape even when some proportion of the habitat is lost. Our proposed method also shows that a landscape with 100% of forest maximizes the functional and phylogenetic diversities of bats (Figure 3).

## DISCUSSION

The method proposed here (“mixture” function), when implemented with replacement of individuals, performed as expected throughout the different tested scenarios and proved to provide reliable curves of diversity in landscapes with different proportions of habitats. The results of the “mixture” function implemented with replacement did not match the predictions of Scenarios 6–8, for *q* = 1 and *q* = 2. However, a close examination shows that this does not indicate a failure of the method. The dominant species in our simulations were defined randomly and differed between assemblages. Therefore, when we combine both assemblages, we obtain a larger number of species with a relevant amount of individuals, increasing the pooled evenness of the sample. This explains why in these scenarios we noticed a higher diversity with intermediate proportions of the two habitats in the landscape when we increased the value of *q*. This situation may not occur in real assemblages, given that abundant species in one habitat are frequently abundant in other habitats as well (Carvalho et al., 2020, 2021; MacIvor & Lundholm, 2011). Indeed, when we run these scenarios with the abundance rank of the species being equal among the two assemblages, our method correctly identifies greater diversity when there are greater amounts of the richer habitat (Appendix S1: Figure S1).

Expectedly, our proposed method correctly identified in our study case that bat species richness (*q* = 0) is maximum when the landscape is composed predominantly by forests, a pattern that is also evidenced in the functional and phylogenetic diversities. For higher order Hill numbers (*q* = 1 and *q* = 2), more weight is given to more common species. Therefore, even less than 50% of forest in the landscape is enough to add common species to the landscape. Furthermore, the occurrence of some soybean plantations in the landscape would increase the pooled evenness of the landscape, resulting in an unchanged diversity in landscapes from intermediate proportions of both habitats to a landscape dominated by forests. Therefore, our method correctly identified the habitat proportions that maximize landscape diversity both in the simulations and in a real dataset.

We tested our method mostly with two habitats, although it can be used with any number of habitats. Nevertheless, carrying out an analysis with more than six habitats can be very computer intensive. Although it is difficult to draw predictions for the more complex scenarios, the simulation with Scenario 1 behaved expectedly when we tested it with three assemblages. Thus, it seems safe to conclude that our method will also provide reliable results in different scenarios with more than two habitats.

The proposed method is related to a previous method proposed by Chao et al. (2019). Indeed, the method proposed by Chao et al. (2019) leads to results similar to those of the function “mixture” applied without replacement (Appendix S1: Figure S2). Besides this aspect, both methods also differ in the number of individuals sampled in each habitat. While the method proposed by Chao et al. (2019) considers one of the assemblages as a “reference sample” and replaces a number of individuals of this sample with the same number of individuals in the other habitat, our method replaces a given proportion of individuals of one habitat with the same proportion of individuals in the other habitat, which is necessary to account for differences in the carrying capacity of the habitats. This second difference explains the differences observed in Scenarios 6, 7, and 9 when comparing the results of the method proposed by Chao et al. (2019) and the function “mixture” applied without replacement (Appendix S1: Figure S2). Differently from our method, the method proposed by Chao et al. (2019) is not intended to identify the proportion of habitats in a landscape that maximizes diversity, and considering the differences in the results, the latter should not be used to reach this goal. Also, applying our method without replacement led to results that did not comply with the predictions in several scenarios (i.e., Scenarios 1–5; Appendix S1: Figure S2). The explanation for this is that sampling without replacement selects all the individuals when there is 100% of one habitat in the landscape, returning the observed richness/diversity. However, when there is a more balanced proportion of two habitats in the landscape, sampling the individuals usually misses some of the rarer species in each habitat, thus underestimating the real richness/diversity. This explains the U‐shape of the diversity curves in the simplest scenarios. In Scenarios 6–10, however, the differences between assemblages are strong enough for this U‐shape pattern to be masked. The implementation of our method with replacement also misses the rarer species, but this occurs with the same probability throughout the whole interval of habitat proportions. Therefore, although this means that the richness/diversity is equally underestimated in the whole interval, the shape of the diversity curves behaved just as expected, which is the desired property of a method aimed at quantifying diversity with different proportions of habitat in the landscape. Therefore, we recommend that the function “mixture” is used only with replacement, which is its default application.

Considering that the abundance of individuals sampled in each habitat affects the results of the “mixture” function proposed here, the sampling effort must be balanced between the two surveyed habitats, to allow a meaningful comparison and so that the recorded differences in the abundances (i.e., differences in the carrying capacity) reflect real differences in the local abundance. Balancing the sampling effort is under the control of the researcher and should be feasible in most cases. However, when the sampling effort is unbalanced, this problem can be circumvented by the rarefaction of the larger sample.

One characteristic of the estimated results with our method is the relatively wide 95% CIs. Thus, although the average estimates behaved as expected, in most cases there was a considerable overlap of the CIs between the maximum and minimum estimates, indicating some degree of uncertainty in which proportion of the habitats maximize species richness/diversity. This reflects the random selection of individuals in the samples. Biological communities do not behave randomly, as the species differ in their response to land use changes (Joyce et al., 2018; Stork et al., 2009). However, we cannot anticipate how the species will react to such changes without proper ecological knowledge about the majority of the species in an assemblage. Thus, in real assemblages, the responses may differ from the central tendency pointed by our method, but still may be within the 95% CIs. This explains why the 95% CIs must be wide. On the other hand, in the more complex scenarios (Scenarios 6–10), which must better represent the characteristics of biological communities, it is possible to observe some point in the curves in which species richness/diversity is clearly lower than in others. Furthermore, when the habitats clearly differ in species richness and diversity (bat study case), our method shows this significant difference, highlighting the robustness of the proposed method to estimate the combined species richness/diversity in landscapes with different habitat proportions. Lastly, the 95% CIs will be lower when there are fewer rare species, given that at least one individual of the more abundant species may be sampled in most runs, making the estimates of richness more similar between runs. Thus, higher sampling effort in the field will reduce the CIs favoring the performance of the method.

## CONCLUSIONS

The function “mixture”, besides presenting a reliable way to project landscapes that maximize taxonomic diversity when there are a number of different habitats, also works with phylogenetic and functional diversities, which is important in the context of maintaining unique lineages and ecosystem services. Despite our method having the caveat of underestimating richness/diversity, this property is equally distributed over all the combinations of habitats in the landscape. Therefore, the identification of the proportions of each habitat that leads to higher biodiversity in the landscape is reliable, which is our main goal. Among other possible situations, our method may be used whenever conservation practitioners are able to choose among different anthropogenic land uses that may be implemented in a landscape (e.g., agricultural systems such as pasture or silviculture), being able to identify which alternative maximizes the diversity (taxonomic, phylogenetic, or functional) in the landscape. Another possibility is to identify the maximum amount of a landscape that may be converted to economic activities without causing significant biodiversity loss. The use of the method is straightforward, and in its simplest form (taxonomic diversity, *q* = 0), conservation practitioners may rely on the default options and provide only a habitats × species matrix. Applying the method with different *q* values (e.g., *q* = 1 and *q* = 2) for taxonomic diversity is also straightforward, since it only demands an additional argument in the function. Assessing phylogenetic and functional diversity may be more challenging for practitioners, as phylogenetic or functional data not only are often hard to obtain, but also demand advanced skills for their manipulation. In such cases, we encourage practitioners and data scientists to collaborate, in addition to experts in the taxa being used for analyses. Multidisciplinary collaborations are important to avoid oversimplification of methods leading to crucial conservation decisions.

Global land use change with consequent modifications in landscape composition not only is widespread but also shows no signs of reaching a plateau. This has consequences not only for biodiversity but also for the ecosystem services on which we depend. In this context, the method proposed here is a reliable and important tool for landscape planning, helping to project landscapes that conciliate economic activities and conservation value.

## AUTHOR CONTRIBUTIONS

Renato Richard Hilário, William Douglas Carvalho, and Pedro Cardoso have participated in the conceptualization of the study. Renato Richard Hilário and Pedro Cardoso have carried out the formal analysis, code development, and manuscript writing. William Douglas Carvalho and Bruna Da Silva Xavier provided the data for the analysis. Jorge M. Palmeirim, Marcus Vinícius Vieira, and Karen Mustin have contributed with ideas and reviewing the manuscript.

## CONFLICT OF INTEREST STATEMENT

The authors declare no conflicts of interest.

## Supporting information


Appendix S1.

## Data Availability

Data are available in Figshare as follows: simulation assemblages (Hilário, 2023c), https://doi.org/10.6084/m9.figshare.24573454; case study data (Hilário, 2023a), https://doi.org/10.6084/m9.figshare.24573445. Code is available as follows: function “mixture” (Hilário, 2023b) in Figshare at https://doi.org/10.6084/m9.figshare.24717897; package BAT (Cardoso, Mammola, et al., 2024) in CRAN at https://doi.org/10.32614/CRAN.package.BAT.
