# Supplementary material for: A new tool to quantify biodiversity change under landscape transformation
Source: Ecol Appl. 2024 Dec 5;35(1):e3071. doi: 10.1002/eap.3071 (PMC11725996; doi:10.1002/eap.3071)
Supplement: Supplementary file 1 — Appendix S1. [file EAP-35-e3071-s001.pdf]

Appendix S1

Ecological Applications

A new tool to quantify biodiversity change under landscape transformation

Renato Richard Hilário, William Douglas Carvalho, Bruna Da Silva Xavier, Jorge M.

Palmeirim, Marcus Vinícius Vieira, Karen Mustin, Pedro Cardoso

FIGURE S1 Results of the scenarios 6, 7, and 8 when the abundance rank among assemblages are equal. The columns represent the results of the function 'mixture' (described in this study) with replacement for q=0, q=1 and q=2. The shaded areas represent the 95% confidence intervals.

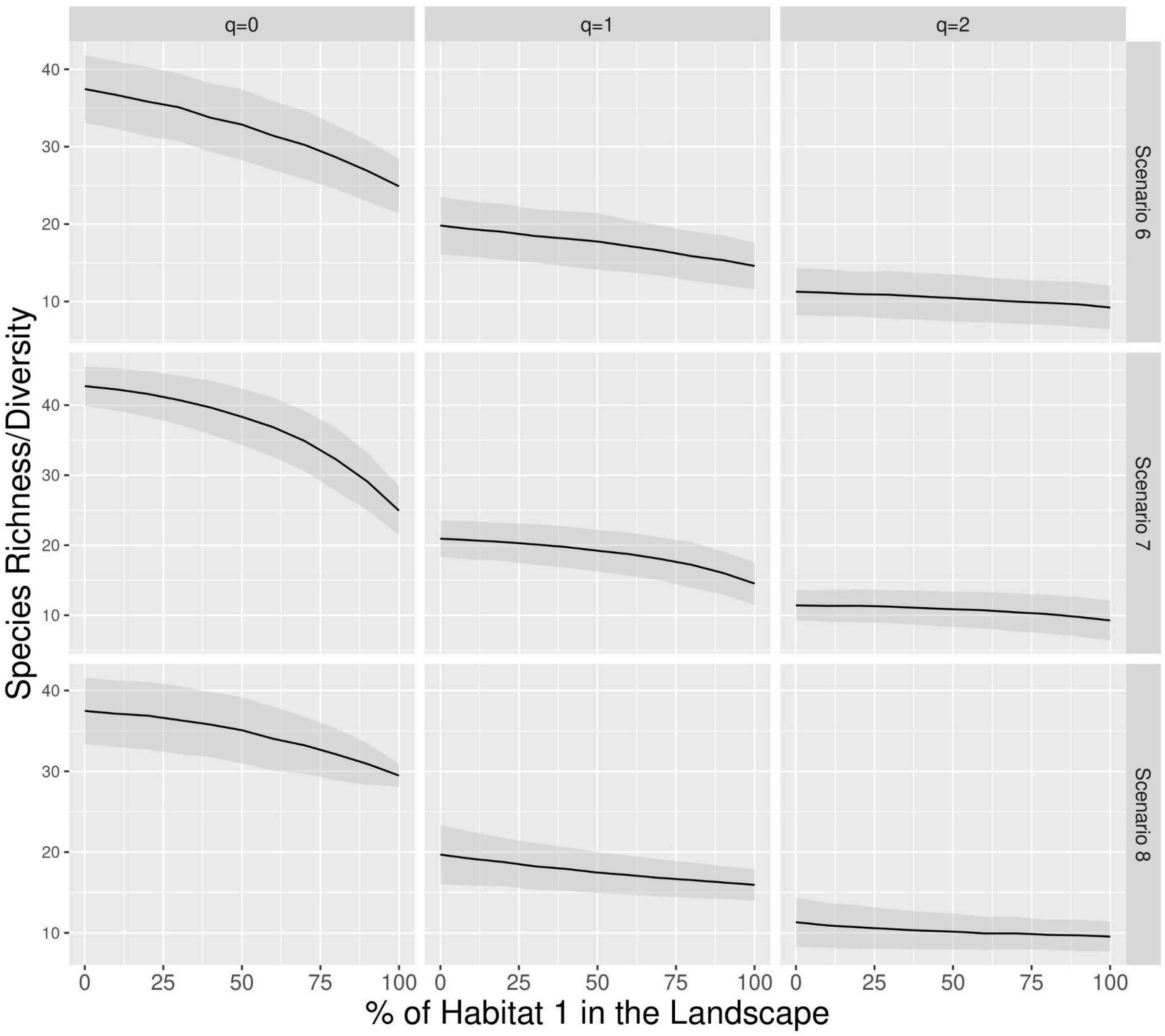

FIGURE S2 Variation of species richness according to different proportions of original habitat (which hosts the first assemblage as described in Table 1) in the landscape in the ten tested scenarios (rows). The first two columns represent the results of the function 'mixture' (described in this study) with replacement and without replacement, respectively. The shaded areas represent the 95% confidence intervals. The third column shows the results of the function 'Abundance' (Chao et al., 2019). The results refer only to q=0.

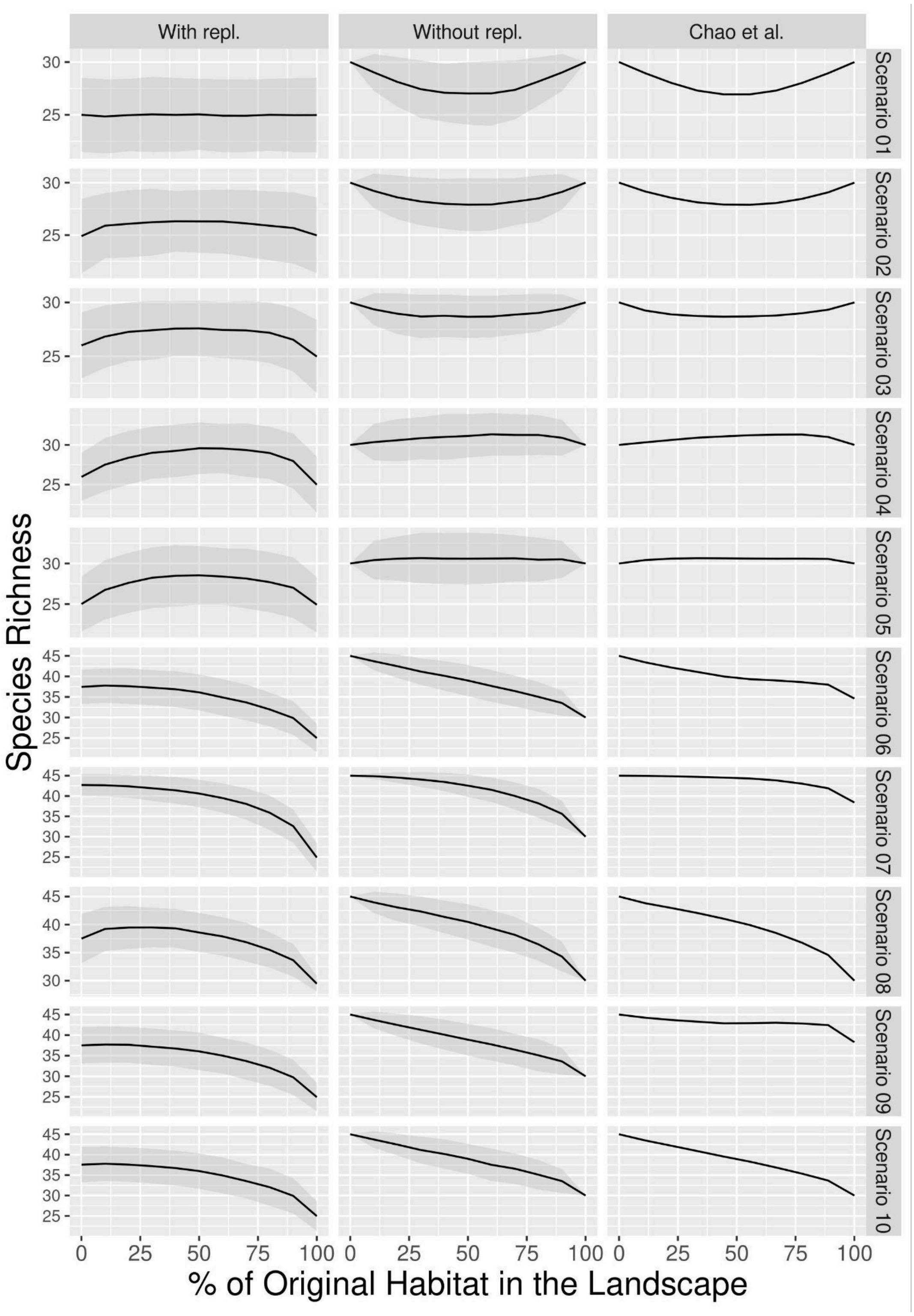

Reference: Chao, A., Colwell, R. K., Gotelli, N. J., & Thorn, S. (2019). Proportional mixture of two rarefaction/extrapolation curves to forecast biodiversity changes under landscape transformation. Ecology Letters, 22(11), 1913-1922. https://doi.org/10.1111/ele.13322
